# A group version of the OurRelationship program within residential substance use treatment: preliminary evidence for improving responses to romantic relationship conflict for those dealing with substance misuse

**DOI:** 10.3389/fpsyg.2024.1307640

**Published:** 2024-12-19

**Authors:** Gery C. Karantzas, Petra K. Staiger, Daniel A. Romano, Ashlee Curtis, Courtney Bruscella, Peter G. Miller, Stefan Gruenert, John W. Toumbourou, Brian D. Doss, Andrew Christensen

**Affiliations:** ^1^School of Psychology, Deakin University, Geelong, VIC, Australia; ^2^Odyssey House Victoria, Richomond, VIC, Australia; ^3^Department of Psychology, University of Miami, Coral Gables, FL, United States; ^4^Department of Psychology, University of California, Los Angeles, Los Angeles, CA, United States

**Keywords:** substance misuse, relationship problems, OurRelationship, conflict, affect

## Abstract

The aim of this study was to pilot an adapted version of an online relationships program with residents in treatment for alcohol and other drugs (AOD). The OurRelationship (OR) Program, which is based on Integrative Behavioral Couples Therapy, was piloted in a group-based format to determine whether residents' participation in the program would result in decreases in residents' destructive responses and increases in constructive responses to relationship conflict scenarios as well as reductions in negative affect experienced during these conflict scenarios. Residents (*N* = 104) across six residential facilities participated in the pilot over 4 weeks. Pre and post program completion, residents read three vignettes in which they were asked to imagine themselves as experiencing each of the three relationship conflicts with a romantic partner. After reading each vignette, residents completed assessments related to their constructive and destructive behavioral responses to each vignette as well as the degree to which they experienced negative affect. Pre-post comparisons across the three vignettes revealed residents reported reductions in destructive behavioral responses and anger (*d*s −0.31 to −0.58) as well as increases in constructive responses (*d*s 0.33–0.77). The findings provide preliminary evidence regarding the application of programs to address the relationship difficulties experienced by those in AOD treatment. Future research into control trials of the OR program within the AOD sector can help to establish the effectiveness of relationship programs in helping to address the relationship problems of this vulnerable population.

## A group version of the OurRelationship program improves responses to relationship conflict in individuals within substance use residential treatment programs

Destructive conflict patterns have consistently been associated with negative relational consequences (Woodin, 2011). Approximately 30–62% of couples experience relationship dysfunction such as high levels of conflict, relationship dissatisfaction, and/or relationship breakdown (e.g., Doss et al., 2004; Qu et al., 2022). This risk can increase significantly for those who experience problems with substance use (i.e., alcohol, overuse of prescription drugs, or illicit substances; e.g., Edwards et al., 2018; Janota et al., 2024). Problematic use of alcohol and other drugs (AOD) perpetuates romantic relationship conflict (Farrelly et al., 2019) by interfering with peoples' capacity to perspective take, problem-solve, and empathize during conflict interactions (e.g., Kane et al., 2024; Schmidt et al., 2016). Furthermore, substance misuse has a disinhibitory component by disrupting executive functioning and self-regulatory processes. These processes are implicated in the inhibition of aggressive tendencies and impulses as well as the ability to accurately perceive threat and other social cues that can drive physical and psychological abuse (e.g., Parrott and Eckhardt, 2018). As a result, many people who experience problems with substance misuse are unable to resolve their relationship issues without specialized support, and unlikely to seek help if concerned about being subject to negative judgments and shaming because of their substance misuse.

Relationship education and therapy for individuals and couples experiencing substance use issues may help them navigate relationship problems, their ability to engage in constructive relationship patterns, and decrease destructive patterns (Kane et al., 2024; Karantzas et al., 2023; Song et al., 2023). Indeed, meta-analyses highlight that couples therapy tailored to substance use (such as Behavioral Couples Therapy) demonstrates a moderate effect size in reducing partner conflict and alcohol consumption (Schmit et al., 2022). However, couples therapy typically requires face-to-face weekly sessions with a trained therapist. This method of delivery severely limits accessibility (i.e., travel and cost), necessitates both partners' involvement, and requires an available and sufficiently trained workforce to deliver therapy. Innovations in digital technologies have yielded highly flexible, accessible, and effective relationship programs that can be delivered online to individuals (Nowlan et al., 2017) or couples (Doss et al., 2016, 2020). Furthermore, these programs provide feedback and strategies tailored to a person or couple's relationship issues. For example, the OurRelationship (OR) program (Doss et al., 2016, 2020) targets people who experience relationship difficulties, often co-occurring with problematic substance use. Specifically, community trials of the OR program found that for low-income individuals and couples, participating in the program resulted in reductions in alcohol use up to 1 year post program completion (Roddy et al., 2021). However, the program has not been trialed with individuals currently seeking treatment for chronic substance misuse.

The OR program is based on Integrative Behavioral Couple Therapy (Christensen and Jacobson, 1998; Christensen et al., 2020) and is a self-directed online program that is completed individually by one or both members of the couple. The program comprises of 8 h of education/activities divided into three sections: (1) Observe, (2) Understand, and (3) Respond. In the *Observe* section, individuals answer questions and receive personalized feedback to help them identify the core issues of concern. In the *Understand* section, individuals engage in activities to achieve a more accurate understanding of their relationship, focusing on their: differences and similarities, emotional experiences, external stressors, and patterns of communication and conflict. In the *Respond* section, participants engage in strategies to respond to conflicts and recover from it, balancing acceptance with self-change.

### The current study

The aim of this study was to pilot an adapted version of the OR program within AOD residential facilities and to investigate whether the OR program would improve the conflict responses of those in treatment for substance misuse. In its original form, the OR program is completed by an individual or couple about their current relationship. For the current study, two key adaptations were made. First, residents undertake the program in a group-based format as clinician and resident feedback from participating facilities indicated that group discussion would be integral to residents' abilities to share, perspective take, and integrate key learnings. Second, because 80–90% of residents were not in a current romantic relationship, participants not in a current relationship were asked to draw on a recent significant past romantic relationship that was at least of 6 months duration.

In the current study, we examined whether the program would result in pre-to-post changes in residents' behavioral responses to relationship conflict scenarios. This is because the OR pilot included residents who were not in a current relationship. Furthermore, the use of vignettes features widely in both substance use and relationship research to assess attitudes, affect, decision-making, and relationship behaviors (e.g., Ashford et al., 2018; Verhofstadt et al., 2020). However, we do acknowledge that self-reported responses to imagined scenarios are merely a proxy, and not direct evidence for how people would actually behave when faced with these same situations in real life (Eifler and Petzold, 2019). Furthermore, our study employed a pre-post design rather than a control group design because management across our residential treatment sites requested that we do not have controls groups given the therapeutic residential environment. This is because all our residential sites operated within therapeutic communities framework (De Leon et al., 2021). This approach to treatment fosters cohesive communities where residents have a significant involvement in decision-making and day-to-day responsibilities within their facility. As part of this framework, clients discuss their therapeutic group work in their daily debriefing sessions. Thus, it would be unethical to ask clients to keep the skills and knowledge they have learnt from participating in the OR program to themselves. Given this, if we were to employ a control group, then control group participants would be exposed to aspects of the program through their interaction with OR participants, and therefore contaminating any “true” control effects (Magill et al., 2019).

Given our pre-post design, we expected participation in the program to be associated with decreases in destructive responses and increases in constructive responses in hypothetical situations of relationship conflict (hypothesis 1). Furthermore, because relationship conflicts often create negative affect (Overall et al., 2015), and the OR program focuses on understanding and dealing with the emotions that are triggered by relationship conflicts (Doss et al., 2013), we were also interested in assessing whether participants would demonstrate decreases in negative affect when exposed to relationship conflict scenarios. We focused on two of the most common emotions experienced in relationship conflicts—anger and anxiety (Overall et al., 2015)—and expected that participation in the OR program would be associated with decreases in both emotions when faced with relationship conflicts (hypothesis 2).

## Method

### Participants

Participants were 104 clients [cisgender males = 61 (59%); cisgender females = 42 (40%); non-binary = 1 (1%]) aged 21–62 years (*M* = 37.00, *SD* = 9.90) undertaking treatment for substance use across six therapeutic community residential facilities. Of the 104 that commenced, 88 completed the post-assessment, resulting in 15% attrition. The majority of the sample (86%) were of Anglo Saxon/European background and 6% were of Indigenous heritage. Only 7% of clients were currently in a romantic relationship; thus, most clients focused on a past romantic relationship. The average current or past romantic relationship length was 6.14 years (*SD* = 5.67) and 20% were currently in a committed relationship (i.e., married, engaged, and defacto) while 51% had been in a committed relationship in the past. The remainder of the sample were currently dating (or had experience with dating in the past). Over 74% of participants were the victims of at least one act of emotional abuse over the last 6 months in either their current or past relationship, while 74% had perpetrated one act of emotional abuse within this same timeframe (in a current or past relationship). Furthermore, 78% had experienced as well as perpetrated at least 3 acts of physical assault in either their current or past relationship. Prior to commencing the OR program, participants evidenced mild depression^1^ (*M* = 9.73, *SD* = 8.56) and anxiety (*M* = 7.44, *SD* = 7.21), and reported stress levels well-above the normal Australian range (*M* = 13.05, *SD* = 7.48; see text footnote 1). Eligibility for entry into the residential services required a diagnosis of Substance Use Disorder. Drug type dependence at entry into the residential services varied across participants with 60% noting amphetamines, 49% alcohol, 21% opiates, 36% cannabis, and 25% for unprescribed benzodiazepines.^2^

### Materials and procedure

Residents were informed about the program by the clinicians at the residential facility and instructed to express their interest to participate in the program to facility clinicians. All clients interested in the program were then screened for their eligibility to participate based on the following inclusion/exclusion criteria: (1) clients needed to be at least 18 years of age, (2) fluent in English, (3) had a significant romantic relationship of at least 6 months in duration that would be their *relationship of focus* during the intervention, (4) not in a current relationship in which there was evidence of family violence (either self-reported, or assessed upon intake by the residential clinical team and then discussed with the research team), (5) experiencing high emotional instability as assessed by their treating clinician. Eligible residents were invited to an introduction session where they watched a video about the program. Interested participants read an information sheet about the study, provided written informed consent, and completed a baseline questionnaire before commencing the program the following week. Ethics approval for the study was provided by the University Human Research Ethics Committee (DUHREC: 2019-195). The study was not pre-registered.

#### Adaptation of the program for AOD residential facilities

Our group-based approach was delivered across six different therapeutic community residential facilities. Facilities that follow a therapeutic communities framework provide a structured setting in which residents take on various roles and responsibilities for running the community they are part of within the facility (e.g., cooking, cleaning). Residents also undertake individual and group-based work related to substance misuse, mental health, social and life skills (Kowalchuk and Reed, 2012). As part of the social skills work, peer relationship issues are addressed, but the topic of romantic relationships is not covered.

Each group that participated in the OR program comprised of between 6 and 10 residents. The groups occurred weekly for 4 weeks. Two group facilitators supported the delivery of the program, but each group member completed the online material individually. At key points in the program—such as when completing a self-reflective activity—the group came together for a discussion led by the program facilitators. The program delivery was manualized to ensure consistency of delivery and facilitators received weekly supervision by a senior clinician. Each of the 4 weekly sessions ran for 3 h. Therefore, the total program time was 12 h. The group-version of the program was 4 h longer than the original version to allow additional time (1 h per session) for the facilitated group-based discussions that accompanied the completion of self-reflective activities. All participants attended all program weeks and completed all activities across the four sessions.

#### Vignettes

Before and after the OR program, participants were presented with vignettes of couple interactions involving issues of substance use and asked to imagine themselves in three situations: being confronted by an angry partner after a night of binge drinking (vignette 1), a partner expressing mistrust of the participant's ability to abstain from using substances (vignette 2), and being confronted by an angry romantic partner after taking a day off from work as a result of the participant's escalation of substance use (vignette 3) (see Supplementary Table A). The vignettes were developed through a co-design process with staff (*n* = 4) and residents [*n* = 4 (not involved in the pilot)]. These vignettes were then piloted with a further three staff and three residents not involved in the study. This additional piloting was sought to enhance the ecological validity of the vignettes, such that they represented situations that were deemed as representative of the relational conflicts that ensue regarding substance misuse. The order of the presentation of the vignettes was counterbalanced across all participants.

#### Pre-post vignette assessments

After reading the three vignettes, participants completed two measures assessing their emotional and behavioral responses to each vignettes. These measures are outlined below.

##### Negative affect

Participants were assessed on the levels of anger and anxiety they experienced in response to the situation presented in each vignette. Assessments of anger (four items, α = 0.82; ω = 0.81) and anxiety (four items, α = 0.75; ω = 0.78) were drawn from the International Positive and Negative Affect Schedule (PANAS) Short Form (Thompson, 2007; Karantzas and Kambouropoulos, 2019). All items were rated on a 5-point scale ranging from 1 (*A little*) to 5 (*A lot*). The correlation between the two negative affect scales was *r* = 0.55.

##### Behavioral responses

Participants were presented with 15 items that were adapted from the Romantic Partner Conflict Scale (Zacchilli et al., 2009; Karantzas and Kambouropoulos, 2019) assessing their behavioral responses to the vignette (e.g., “Tell my partner openly that I disagree,” “Shove or push my partner if they continue to criticize or provoke me,” “Act in a calm way”). Items were rated on a 7-point scale ranging from 1 (*Extremely unlikely that I would react like this*) to 7 (*Extremely likely that I would react like this*). The items comprised two subscales relating to destructive responses (i.e., responses that escalate the conflict and do not resolve an issue, 10 items, α = 0.86, ω = 0.87) and constructive responses (i.e., responses that attempt to reduce conflict and seek conflict solution, five items, α = 0.83, ω = 0.84). The correlation between the two behavioral response scales was *r* = −0.37.

### Data analysis

To examine changes in negative affect and behavioral responses to the vignettes after taking part in the program, two repeated multivariate analysis of variance (MANOVA) were conducted. The first MANOVA examined differences from pre to post program across two negative affect dependent variables –anger and anxiety. The second MANOVA examined differences from pre to post program across two dependent variables, destructive responses and constructive responses. *Apriori* power was estimated using Gpower 3.1 (Faul et al., 2007) for conducting repeated measures MANOVA [*f* = 0.15, α = 0.05 (two-tailed), repeated assessment *r* = 0.5]. The estimated sample size necessary to achieve a power of 0.80 was 90 participants.

To determine whether psychological distress and past experiences of relationship abuse may affect our findings, we conducted a series of preliminary analyses (i.e., Multivariate Analysis of Covariance) including these control variables as covariates (data and analytic code are available by contacting the corresponding author).

## Results

Our preliminary multivariate analysis of covariance tests indicated that psychological distress and relationship abuse were not statistically significant predictors of outcomes. Given this, and to increase ease of interpretation of findings, we only present the results of our analyses excluding these covariates (i.e., MANOVAs).

### Behavioral responses

Significant multivariate main effects were found for behavioral responses from pre to post program for all three vignettes [chronic drinking vignette: *Pillai's Trace* = 0.27, *F*_(2, 86)_ = 16.20, *p* < 0.001; abstinence vignette: *Pillai's Trace* = 0.24, *F*_(2, 86)_ = 13.84, *p* < 0.001; escalation vignette: *Pillai's Trace* = 0.32, *F*_(2, 86)_ = 20.01, *p* < 0.001]. As shown in Table 1, participants reported a reduction in destructive responses [chronic drinking vignette: *F*_(1, 87)_ = 16.18, *p* < 0.001; abstinence vignette: *F*_(2, 86)_ = 22.07, *p* < 0.001; escalation vignette: *F*_(1, 87)_ = 11.79, *p* = 0.001] and an increase in constructive responses [chronic drinking vignette: *F*_(1, 87)_ = 19.65, *p* < 0.001; abstinence vignette: *F*_(1, 87)_ = 14.62, *p* < 0.001; escalation vignette: *F*_(1, 87)_ = 36.13, *p* < 0.001] after program completion.

**Table 1:** Descriptive statistics, and pre-post effect sizes for emotional and behavioral responses to the vignettes.

**Vignette**	**Variable**	**Pre-program (*****N*** = **104)**			**Post-program (*****N*** = **88)**			***d***
		***M***	***SD***	**Response range**	***M***	***SD***	**Response range**	
Drinking	Anger	2.71	1.00	1–5	2.41	0.94	1–5	−0.31^**^
	Anxiety	3.33	1.10	1–5	3.14	1.13	1–5	−0.17
	Constructive response	3.69	1.24	1–7	4.10	1.25	1–7	0.33^***^
	Destructive response	2.24	1.22	1–6.10	1.75	0.77	1–4.60	−0.47^***^
Abstinence	Anger	2.62	1.13	1–5	2.35	1.03	1–5	−0.25^*^
	Anxiety	2.67	1.15	1–5	2.21	0.94	1–5	−0.43^**^
	Constructive response	3.97	1.39	1–7	4.78	1.46	1.20–7	0.57^***^
	Destructive response	2.40	1.21	1–6	1.80	0.79	1–4.70	−0.58^***^
Escalation	Anger	2.89	1.13	1–5	2.62	1.06	1–5	−0.25
	Anxiety	3.20	1.16	1–5	3.06	1.17	1–5	−0.12
	Constructive response	3.59	1.21	1–7	4.57	1.35	1.20–7	0.77^***^
	Destructive response	2.45	1.36	1–6.30	1.95	0.91	1–5.22	−0.43^**^

^*^p < 0.05.

^**^p < 0.01.

^***^p < 0.001.

### Negative affect

A significant multivariate main effect was found for negative affect from pre to post program for the chronic drinking vignette [*Pillai's Trace* = 0.09, *F*_(2, 86)_ = 4.21, *p* = 0.02] and the abstinence vignette [*Pillai's Trace* = 0.11, *F*_(2, 86)_ = 5.22, *p* = 0.007], but not for the escalation of use vignette [*Pillai's Trace* = 0.04, *F*_(2, 86)_ = 1.70, *p* > 0.05]. As shown in Table 1, in relation to the chronic drinking vignette, only feelings of anger [*F*_(1, 87)_ = 8.29, *p* = 0.005] were significantly lower after having completed the program. In relation to the abstinence vignette, both anger [*F*_(1, 87)_ = 5.59, *p* = 0.02] and anxiety [*F*_(1, 87)_ = 9.95, *p* = 0.002] were lower after having completed the program (see Table 1).

## Discussion

Our findings provide the first evidence that the group version of the OR program, when adapted for use in substance use residential settings, has the potential to shift resident's behavioral and affective responses in situations of relationship conflict (albeit hypothetical scenarios). Our findings provide preliminary evidence that focusing on enhancing awareness and understanding of the factors that contribute to the difficulties experienced in a current or past romantic relationship, as well as developing constructive strategies to respond to relationship problems, can be beneficial for this population. Our piloting of the vignettes indicated that clients found the vignettes to be scenarios reflective of typical and challenging relationship conflicts for this group of people. This is consistent with findings that strongly implicate relationship conflict as a trigger for the escalation of substance use (Radcliffe et al., 2019). Hence, interventions that can improve ways of dealing with conflict are critical in ensuring positive outcomes for those in treatment for substance use disorders (Kane et al., 2024; Song et al., 2023).

In support of our first hypothesis, reductions in destructive responses and increases in constructive responses were evidenced in all three scenarios at post program completion (compared to pre-program commencement). Furthermore, our findings suggest that the program may help prevent residents from interacting with partners in a manner that is spiteful, conveys partner dissatisfaction, annoyance and entails physical aggression such as pushing, shoving, and hitting. These findings demonstrate that educating residents about constructive patterns of responding to conflict, as well as understanding the reactions of partners, can increase the likelihood that residents can calmly and openly communicate their point of view while also trying to see the partner's point of view and work toward mutual understanding/compromise.

In partial support of our second hypothesis, residents reported reductions in responding with anger in two out of the three imagined scenarios, including partner mistrust regarding abstinence, and responding to a partner's anger after a night of binge drinking. Residents also reported reductions in experiencing anxiety in response to the scenario centered around partner worry and mistrust regarding abstinence. This shift in anxiety is common for those in substance use treatment and may speak to the focus that the OR program places on understanding felt emotions, which includes identifying situations that trigger emotions and understanding why particular emotions are experienced (Christensen et al., 2023; Christensen et al., 2020; Doss et al., 2016, 2020).

Importantly, our pilot demonstrated that relationship gains can be made within a population (at least in forecasting how residents would respond to imagined relationship scenarios) characterized by high levels of relationship conflict and relationship abuse. Moreover, our adaptations to the program (i.e., group format; drawing on a past significant romantic relationship when not involved in a current relationship), appear to maintain the positive benefits of the OR program (see Doss et al., 2016, 2020). Therefore, our implementation of the OR program to the substance use sector is likely to address an important and unmet need, that is, delivery of relationship programs that target the difficulties experienced by those that have been affected by substance misuse.

### Limitations and future directions

There are limitations to our pilot study. First, our study reports on outcomes of resident's responses to imagined relationship scenarios, and as such cannot speak to the resident's actual behavior when faced with relationship conflict. However, our vignettes were developed through a co-design process with staff and residents. Nevertheless, future research should conduct post-program follow-up that assess the affective responses and relationship behaviors of residents as they enter and navigate actual relationships post-residential treatment. Second, although our findings suggest that the OR program improved the conflict and affective responses of residents when faced with scenarios of relationship conflict, we cannot rule out social desirability effects. That is, it is challenging to determine whether participants behavioral and affective responses to vignettes after participating in the OR program somewhat reflected desired responses given exposure to program materials. Therefore, it may be useful for future research to measure residents' tendency for biased responding and to control for this when analyzing results. Third, our pilot does not include a control group; thus, we are unable to comment on whether some of the improvements observed in this study are due to broader substance use treatment rather than to the OR program. Similarly, without a control group or condition in which the program was implemented in its original (individual format), we are unable to determine whether the group-based component of the OR adaptation contributed to the findings above and beyond the OR program itself. Although the inclusion of a control group in this study was both ethically and practically unfeasible given the therapeutic communities approach to treatment used by the residential facilities, future research should conduct control trials in residential facilities that employ treatment approaches that can facilitate RCT designs. The design and conduct of RCTs can enhance the robustness of the evidence-base for the use of the OR program (and our group-based adaptation) within substance use residential facilities. Fourth, for those people who were not currently in a relationship, they were asked to recall and focus on their most recent significant relationship. Although participants were able to complete the program drawing on a past relationship, future efforts would need to further validate this adaptation. Finally, the majority of the sample was Anglo-Saxon, thus future research would benefit from surveying a higher proportion of clients from indigenous and ethnically diverse groups.

## Conclusion

The findings of this study provide important preliminary evidence regarding the merit and application of programs to address the pervasive relationship difficulties experienced by those in treatment for substance use disorders. Future efforts to conduct fully-fledged control trials of the OR program and alike within the sector are important steps in understanding the effectiveness of such programs in addressing the relationship needs of this vulnerable population.

## Data Availability

The datasets presented in this article are not readily available because, the data contains sensitive information with regards to clients within residential treatment facilities. Requests to access the datasets should be directed to: gery.karantzas@deakin.edu.au.

## Footnotes

^1^Sample means reflect mild depression and anxiety and stress above the normal Australian range. Australian adult normative data on the DASS-21: Depression (*M* = 5.14), Anxiety (*M* = 3.48), and Stress (*M* = 7.98; Crawford et al., 2011).

^2^Substance misuse percentages exceed 100% as some residents report misuse across more than one substance.
