# Supplementary material for: A group version of the OurRelationship program within residential substance use treatment: preliminary evidence for improving responses to romantic relationship conflict for those dealing with substance misuse
Source: Front Psychol. 2024 Dec 19;15:1307640. doi: 10.3389/fpsyg.2024.1307640 (PMC11693445; doi:10.3389/fpsyg.2024.1307640)
Supplement: Supplementary file 1 [file Table_1.DOCX]

**Supplemental Material**

Table A

*Vignettes*

| **Vignette 1 (Chronic Drinking)** |
| --- |
| Imagine that you work 5 days a week and that you head out for drinks with your work friends on most days after work. Last night you went out with your work friends and you had too much to drink. You get up the next morning and your head feels foggy. You don’t quite remember how last night ended, but your partner is really upset with you. You try to talk to your partner to find out what’s going on. They give you the cold shoulder and angrily say that they do not want to talk to you because you are such a pathetic loser. |
| **Vignette 2 (Abstinence)** |
| Imagine that it has been about 4 weeks since you have used drugs or alcohol. You are proud of the changes you have made, but you have found not using really challenging. Each time in the past few weeks that you have told your partner you will be catching up with friends, they ask you questions about who you are going out with, if you will be using, and asks what time you will be home. Last night you went out with friends, your partner said to you that they find it hard to trust you and that you will end up using again. |
| **Vignette 3 (Escalation of Use)** |
| Imagine that your use has been escalating and you have been taking more sick days from work to cope with the morning after. Your partner returns home from work, and is frustrated to see you at home again, and knows why you took the day off. Your partner gets angry and says that you are not contributing to the household and that you are selfish. Your partner tells you that they are having to do everything around the house and that you are just a burden. They finish by telling you that they’d be better off without you. |
